# Pseudobacteremia outbreak of biofilm-forming *Achromobacter xylosoxidans* – environmental transmission

**DOI:** 10.1186/s12879-016-1909-0

**Published:** 2016-10-19

**Authors:** Frank Günther, Uta Merle, Uwe Frank, Matthias M. Gaida, Nico T. Mutters

**Affiliations:** 1 Department of Infectious Diseases, Heidelberg University Hospital, Im Neuenheimer Feld 324, D-69120 Heidelberg, Germany; 2 Department of Internal Medicine, Gastroenterology and Infectious Diseases, Heidelberg University Hospital, Heidelberg, Germany; 3 Institute of Pathology, Heidelberg University Hospital, Heidelberg, Germany

**Keywords:** Healthcare-associated infections, Infection control, Biofilm, Environmental cleaning, Disinfection, Tissue dispensers, Achromobacter xylosoxidans

## Abstract

**Background:**

*Achromobacter xylosoxidans* (AX) is known for intrinsic resistance to disinfectants. Our laboratory routine surveillance system detected an unexpected rise in AX bloodstream infections in a 2200-bed hospital. An epidemiological investigation was conducted to find the source and disrupt further transmission.

**Methods:**

Outbreak cases were defined as patients with at least one positive blood culture positive for AX from May 2014 to May 2015. Medical records were reviewed, affected wards, as well as the microbiology laboratory were audited. Additionally, microbiologic culture and biofilm staining for suspected antiseptic reusable tissue dispensers were performed, and isolated AX strains were typed using RAPD PCR and PFGE.

**Results:**

During the outbreak period, AX were isolated from blood cultures from 26 patients. The retrospective cohort study did not reveal common risk factors. The clinical features of the case patients suggested a pseudobacteremia. The reusable tissue dispensers containing Incidin® Plus solution product were found to be contaminated with biofilm-forming AX. Typing of the isolates revealed that blood culture isolates were identical with the strains found in the dispensers.

**Conclusions:**

After changing the usage of the product to single-use and educating staff, the outbreak was terminated. Contamination of dispensers occurred due to insufficient reprocessing, since biofilm disrupting steps were not included in the process.

## Background

*Achromobacter xylosoxidans* (AX) is a well-known pathogen in cystic fibrosis patients but it is rarely seen in other patients [1]. It is, however, widely distributed in nature and its ability to produce biofilm contributes to its high tenacity and survival under unfavourable conditions [2]. Even antiseptics and disinfectants are not always able to eliminate *Achromobacter* spp. [3]. In January 2015 we experienced a significant increase of AX bloodstream infections (BSI) on one of our medical intensive care units (ICU) in our 2,200-bed hospital, and such infections had been rarely seen in the past (AX isolates in 2012: 3 BSI, 4 in respiratory materials, 4 in wound swabs; in 2013: 0 BSI, 2 in respiratory materials, 8 in wound swabs). We also detected an increase in AX isolations from materials other than blood cultures (respiratory materials, rectal and wound swabs). We immediately started investigating, identified the environmental source and terminated the outbreak.

## Methods

### Clinical setting

Heidelberg University Hospital (HUH) is a 2,200-bed university teaching hospital and one of the largest and most renowned hospitals in Germany. It provides a full range of medical and surgical services, including active programs for solid organ, and bone marrow transplantation. Outbreak cases were defined as patients with at least one positive blood culture positive for AX from May 2014 to May 2015. This rather general definition of outbreak cases was used to increase sensitivity that no possible outbreak case would be missed.

### Tissue dispensers

The tissue dispenser buckets (CELTEX®-wipes; Loftex, Bremen, Germany) contain tissues with Incidin® Plus (Ecolab, Monheim, Germany), a Glucoprotamin based disinfectant (Fig. 2a). They have an intended service time of 28 days. After that they need to be reprocessed and refilled with new tissues. According to the manufacturer’s recommendations, multi-use after reprocessing is allowed. However, single-use is recommended for areas with high infection risk (ICU, hemato-oncology, neonatology, burn units). Reprocessing needs to include 3 steps. 1) Product residues and remaining tissues need to be thrown away and dispenser buckets need to be allowed to dry. 2) Rinse with hot water (>55 °C), let dry again 3) wipe-down disinfection with an alcohol-based disinfectant (provided in form of a wipe by the manufacturer) and let dry again. After completion of this 3-step processing dispensers can be refilled with tissues and used for another 28 days, thus multi-use is allowed.

### Isolation and identification of *A. xylosoxidans* isolates

Isolates were recovered from blood samples incubated in the BACTEC™ FX automated microbial detection system (BD diagnostics, Sparks, USA). After Gram staining, isolates were inoculated on appropriate media and incubated at 37 °C overnight. Isolates recovered from other materials were also inoculated on appropriate media and incubated at 37 °C overnight. Identification of bacteria was done by matrix-assisted laser desorption ionization time-of-flight mass spectrometry as described elsewhere [4]. Susceptibility testing was performed according to EUCAST criteria using the Vitek2 system (bioMérieux Clinical Diagnostics, Marcy l'Etoile, France).

### Epidemiologic investigation

**Fig. 1:**
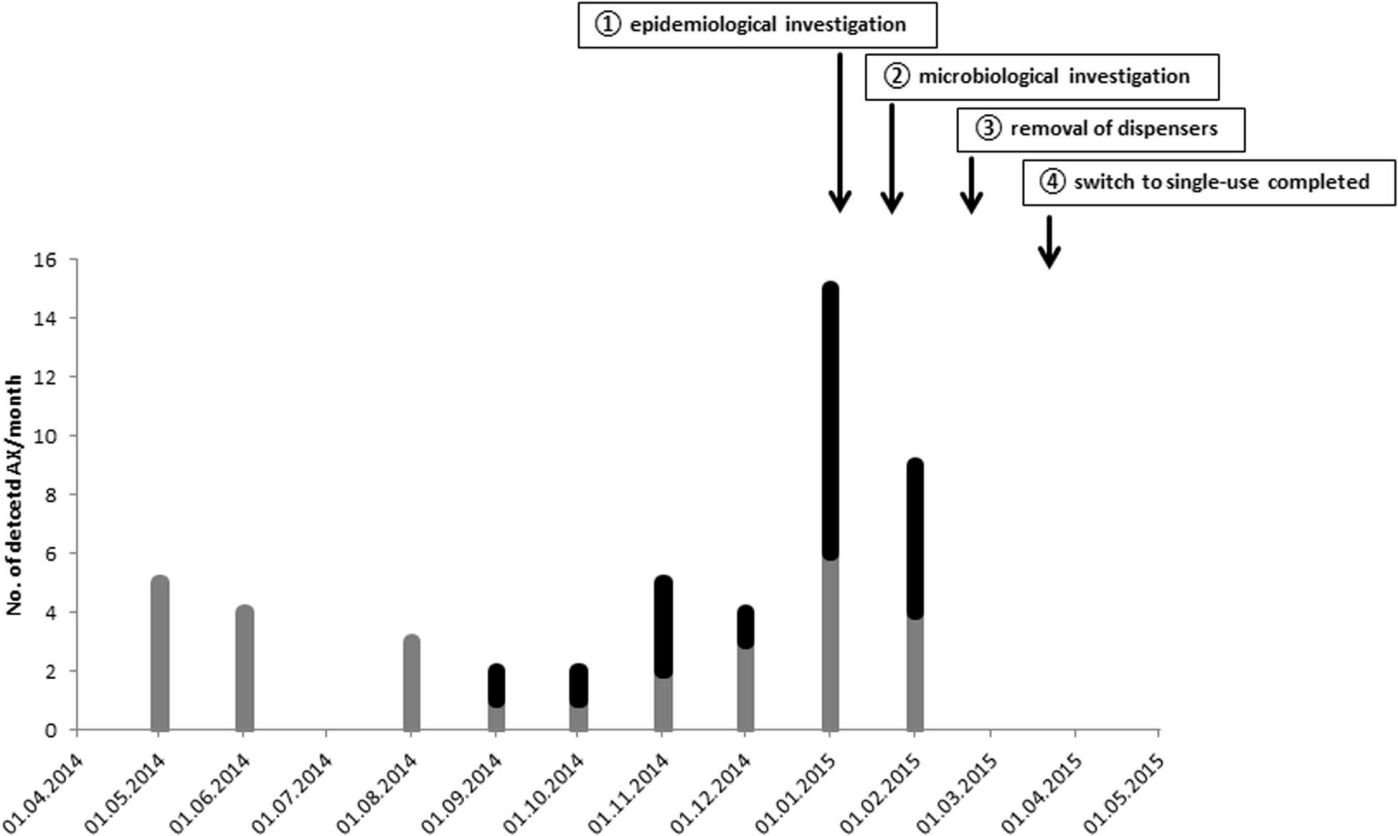
Outbreak chart. AX detected in blood cultures or central venous catheters BSI (*grey*), AX detected elsewhere (*black*) and interventions (*arrows*)

After an increased incidence of cases with a blood culture positive for AX was identified by the microbiological laboratory, an epidemiological investigation was immediately started by the infection control team in the end of January/beginning of February 2015 (① in Fig. 1). A retrospective cohort study to identify common risk factors for AX BSI was conducted. Furthermore, review of all microbiological records to identify undetected cases retrospectively and to determine the baseline BSI rate of AX prior to the suspected outbreak was performed. The patients’ medical records of the additional identified cases were also reviewed to identify common risk factors. The microbiological laboratory was audited to exclude possible contamination of the blood cultures in the laboratory. The identified wards with the majority of the cases were visited and blood culture sampling practices were reviewed. Additionally, the antiseptics and disinfectants used within these wards were checked regarding the sort of products, disinfectant agents, usage patterns of the different wards and possible recent changes of products.

### Microbiological investigation of the reusable surface disinfection tissue dispensers

Upon completion of the epidemiological investigations, the microbiological and molecular investigation followed in February 2015 (② in Fig. 1), including observations of the processing practices of the tissue dispensers. Disinfectant samples were collected from 26 dispensers localized in patient rooms or central service areas. Additionally, two samples were collected from stationary metering units for disinfectants used for refilling the tissue dispensers. Tissues were only tested from dispensers that were found contaminated.

Each sample was tested for bacterial contamination by membrane filtration of 50 ml disinfectant solution trough a 0.45 μm nitrocellulose membrane (Sartorius Stedim, Göttingen, Germany) and rinsed with 250 ml of sodium peptone solution (Merck Millipore, Darmstadt, Germany). Subsequently, the filter was transferred on a Caso-Agar containing LTHTh as neutralizer (Merck Millipore, Darmstadt, Germany). The neutralizer was validated for effectively neutralizing the tested disinfectant agents prior to this study (data not shown). The media were incubated for 5 days at 37 °C and checked for microbial growth every 24 h. Colony forming units (CFU) were counted. If growth was detected, bacteria were identified by MALDI-TOF mass spectrometry (Bruker Daltonik, Bremen, Germany).

### Molecular investigation

All AX isolates were typed using random amplified polymorphic DNA PCR (RAPD PCR) and pulsed field gel electrophoresis (PFGE) (② in Fig. 1). RAPD PCR was performed using primer 272 as described elsewhere [5]. Typing by PFGE of each detected *Achromobacter* isolate was performed using *DraI* as restriction enzyme. Analysis of the gained typing results was performed according to the criteria defined by Tenover et al. [6].

### Analysis of biofilm formation in the contaminated tissue dispensers

To analyze the bacterial habitats in the disposal tissue dispensers, dispensers were collected and possible biofilm formation was tested. Tissue dispenser buckets were screened for bacterial biofilms using a 0.1 % crystalviolet staining solution (Sigma Aldrich, Taufkirchen, Germany). The collected tissue samples were fixed in buffered formalin, embedded in paraffin and subsequently sliced into 4 μm paraffin sections. After deparaffinization with xylene and rehydration in graded alcohols, acridine orange staining (Becton Dickinson, Heidelberg, Germany) was performed to detect biofilms and/or adherent bacteria on singular tissue fibers.

## Results

### Epidemiological investigation

After the routine surveillance system of our laboratory had detected 13 cases of AX BSIs in January and beginning of February 2015 as well as an additional increase in AX isolations from other materials, the epidemiological investigation was started concurrently (① in Fig. 1). Initially, injection of drugs or incorrect handling of intravascular catheters was suspected to cause the BSIs. The performed cohort study, however, did not find any common risk factor to identify the source. When traced back to the wards, most of the cases were found on two connected internal medicine wards: an intensive care unit (ICU) and an intermediate care unit (IMC). Patients are usually transferred from the ICU to the IMC when they clinically improve. Further sporadic cases across the hospital could also be observed, however, no spatial or epidemiological connection between them could be identified. In total, we identified 26 patients from December 2014 to February 2015 with at least one episode of AX bacteraemia; 4 patients had multiple positive blood cultures (Fig. 1). Most of the cases were clinically asymptomatic and did not show symptoms of an infection with AX.

Retrospectively, from 1st May 2014 to 1st May 2015, 49 AX isolates were detected in different clinical specimen of 28 patients of the same clinical unit. Of those, 19 patients were identified with at least one episode of AX bacteraemia, and three patients with multiple blood cultures positive for AX (Fig. 1). The clinical characteristics retrieved from medical records suggested a contamination rather than actual infection with AX.

The audit of the microbiological laboratory revealed that a contamination in the laboratory was very unlikely especially since the spatial distribution of the cases pointed to the medical ICUs. While common risk factors i.e. intravascular catheterization and invasive procedures were often found in all ICU patients, regardless of medical or surgical patients, curiously AX BSI episodes mainly occurred on medical ICUs while the surgical ICUs only had sporadic and very few cases. Since blood culture sampling methods and handling of intravascular catheters did not differ between the surgical and medical department, we suspected a difference in antiseptic use between the departments. While the same surface disinfection and hand disinfection products were used in both departments, only the medical department used reusable surface disinfection tissue dispensers. Those tissues contained Incidin® Plus (Ecolab, Monheim, Germany), a Glucoprotamin based disinfectant used for disinfection of patient-related areas i.e. nightstands and bedrails. Additionally, they were used by the healthcare workers responsible for blood sampling to disinfect the trays where all materials necessary for blood sampling i.e. collection tubes, needles and blood culture bottles were placed on. The reusable tissue dispensers were manually processed supposedly according to manufacturer’s recommendations of every 28 days. However, compliance with and efficacy of manual processing in clinical practice was never assessed. Thus, we started a microbiological investigation of the reusable dispensers and the actual manual processing that was carried out.

### Microbiological and molecular investigations

Incidin® Plus solutions from 23 dispensers were collected and tested (② in Fig. 1). A contamination with AX of at least ≥10^3 CFU per mL was found in 69.6 % (16/23) of the tested dispensers. All tissues tested from contaminated dispensers were also found contaminated with AX. The tested stationary metering units for disinfectants showed no contamination with AX. No other bacteria than AX was detected.

The molecular typing investigations showed, that 7 AX isolates recovered from patients’ blood cultures were indistinguishable from 15 typed AX isolates in the dispensers and could therefore be regarded as part of the cluster type A. One AX isolate showed minimal differences in banding pattern of PFGE and was therefore interpreted as closely related type A1. The typing results showed congruent results for both typing techniques. Two further isolates recovered from blood cultures collected from patients of other clinical units showed different banding patterns in both typing techniques compared to the outbreak isolates and was assigned to type B.

### Infection control interventions

Contaminated dispensers were removed from affected wards starting from 2nd March 2015 (③ in Fig. 1). Additionally, processing of dispensers was terminated and usage of dispensers was only allowed as single use. Measures were implemented until 17th March 2015 (④ in Fig. 1). Within 2 months of follow up, no further episodes of AX bacteraemia or detection of AX in clinical specimens from the affected wards were identified (Fig. 1).

### Analysis of biofilm formation in the contaminated tissue dispensers

**Fig. 2:**
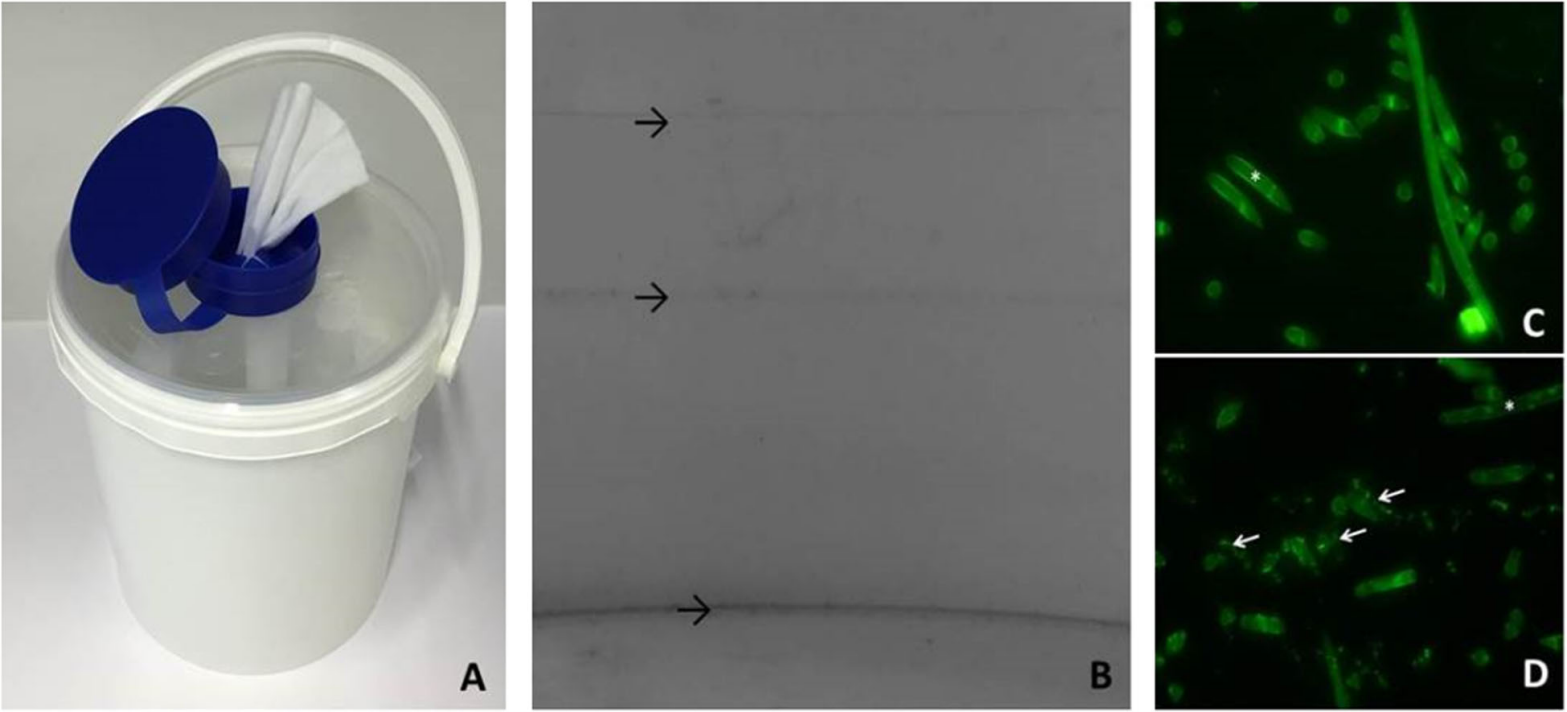
Pictures of dispensers and biofilm stains. **a**: a tissue dispenser bucket. **b**: AX biofilm on wall and bottom of dispenser buckets stained with crystalviolet; *black* arrows: increased biofilm formation on former liquid levels (*upper arrows*) and at the bottom of the bucket (*lower arrow*). **c**: acridine orange stained tissue sample of a newly assembled dispenser **d**: AX biofilm detected by acridine orange staining on tissues collected from a dispenser 5 weeks after reprocessing (*white arrows*: biofilm; * tissue fiber; 200× magnification)

Bacterial biofilms were detected in all reprocessed tissue dispensers within 1 week after completion of reprocessing. Biofilms were detected on buckets (Fig. 2b) as well as on tissues (Fig. 2d). Clearly visible bacterial contamination on tissue fibers was detectable after 5 weeks (Fig. 2d) in reprocessed tissue dispensers. In contrast, newly assembled tissue dispensers that were never reprocessed did not show biofilm formation (Fig. 2c).

## Discussion and conclusions

The pseudobacteremia was caused by contaminated Incidin® Plus tissues that were used to clean patient-related areas and trays for blood sampling. The tissues were also used to wipe off the blood culture bottles after removal of the lid, leading to the contamination of blood cultures and causing the pseudobacteremia. The contamination of tissue dispensers was permitted by breaches in the manual processing of the reusable tissue dispensers. Our investigation revealed that during tissue dispenser processing the same disinfectant was used for cleaning the dispenser buckets as well as in the tissues. Solutions with only one specific active agent, however, may get contaminated and thereby contribute to the transmission of pathogens [3]. Investigating the manual cleaning process of the tissue dispensers at our hospital revealed that no high temperature step or the use of a biofilm-active cleaning agent was used in the cleaning process. Without those essential steps manual or automatic processes must be considered insufficient for prevention of re-/contamination of tissues [7]. The continuous use of the same or similar disinfectant in processing reusable dispensers supports the selection of resistant clonal lineages, which can result in the establishment of resident homogenous bacterial communities in such dispensers. Bacteria in biofilms are then able to adapt to the used disinfectant and survive the reprocessing procedure. Consequently, extensive bacterial contamination of environmental surfaces by standard disinfection procedures is possible [8]. The dispensers contaminated with biofilm-forming Gram-negatives, such as AX, can then become a source of pathogen transmission and possible consecutive infections [9, 10]. Infection control departments should be aware of reusable tissue dispensers and evaluate the internally used processing procedures. In areas where patients are at high-risk for developing an infection (ICU, hemato-oncology, neonatology, burn units, transplant units) tissue dispenser should strictly be used according to manufacturers’ recommendations and preferably as single-use only.

## Abbreviations

AX: Achromobacter xylosoxidans
BSI: bloodstream infections
CFU: colony forming units
HUH: Heidelberg University Hospital
ICU: intensive care unit
IMC: intermediate care unit
PFGE: pulsed field gel electrophoresis
RAPD: random amplified polymorphic DNA PCR
